# Feasibility of keeping Mars warm with nanoparticles

**DOI:** 10.1126/sciadv.adn4650

**Published:** 2024-08-07

**Authors:** Samaneh Ansari, Edwin S. Kite, Ramses Ramirez, Liam J. Steele, Hooman Mohseni

**Affiliations:** ^1^ Department of Electrical and Computer Engineering, Northwestern University, Evanston, IL, USA.; ^2^ Department of the Geophysical Sciences, University of Chicago, Chicago, IL, USA.; ^3^ Department of Physics, University of Central Florida, Orlando, FL, USA.; ^4^ European Center for Medium-Range Weather Forecasts, Reading, UK.

## Abstract

One-third of Mars’ surface has shallow-buried H_2_O, but it is currently too cold for use by life. Proposals to warm Mars using greenhouse gases require a large mass of ingredients that are rare on Mars’ surface. However, we show here that artificial aerosols made from materials that are readily available at Mars—for example, conductive nanorods that are ~9 micrometers long—could warm Mars >5 × 10^3^ time smore effectively than the best gases. Such nanoparticles forward-scatter sunlight and efficiently block upwelling thermal infrared. Like the natural dust of Mars, they are swept high into Mars’ atmosphere, allowing delivery from the near-surface. For a 10-year particle lifetime, two climate models indicate that sustained release at 30 liters per second would globally warm Mars by ≳30 kelvin and start to melt the ice. Therefore, if nanoparticles can be made at scale on (or delivered to) Mars, then the barrier to warming of Mars appears to be less high than previously thought.

## Teaser

Warming Mars with artificial aerosols appears to be feasible.

## INTRODUCTION

Dry river valleys cross Mars’s once-habitable surface (*1*, *2*), but today the icy soil is too cold for Earth-derived life (*3*–*5*). Streams may have flowed as recently as 600 thousand years ago (*6*), hinting at a planet on the cusp of habitability. Many methods have been proposed to warm Mars’ surface by closing the spectral windows, centered around wavelengths (λ) 22 and 10 μm, through which the surface is cooled by thermal infrared radiation upwelling to space (*7*–*9*). Modern Mars has a thin (~6 mbar) CO_2_ atmosphere that provides only ~5 K greenhouse warming via absorption in the 15-μm band (*10*), and Mars apparently lacks enough condensed or mineralized CO_2_ to restore a warm climate (*11*). The spectral windows can be closed using artificial greenhouse gases (e.g., chloroflourocarbons) (*8*, *12*), but this would require volatilizing ~100,000 megatons of fluorine, which is sparse on the Mars surface. An alternative approach is suggested by natural Mars dust aerosol. Mars dust is almost all ultimately sourced from slow comminution [indirect estimate *O*(3) liters/s (*13*)] of iron-rich minerals on Mars’ surface. Because of its small size (1.5-μm effective radius), Mars dust is lofted to high altitude (altitude of peak dust mass mixing ratio, 15 to 25 km), is always visible in the Mars sky, and is present up to >60 km altitude (*14*–*15*). Natural Mars dust aerosol lowers daytime surface temperature [e.g., (*16*)], but this is due to compositional and geometric specifics that can be modified in the case of engineered dust. For example, a nanorod about half as long as the wavelength of upwelling thermal infrared radiation should interact strongly with that radiation (*17*).

## RESULTS

**Fig. 1.**
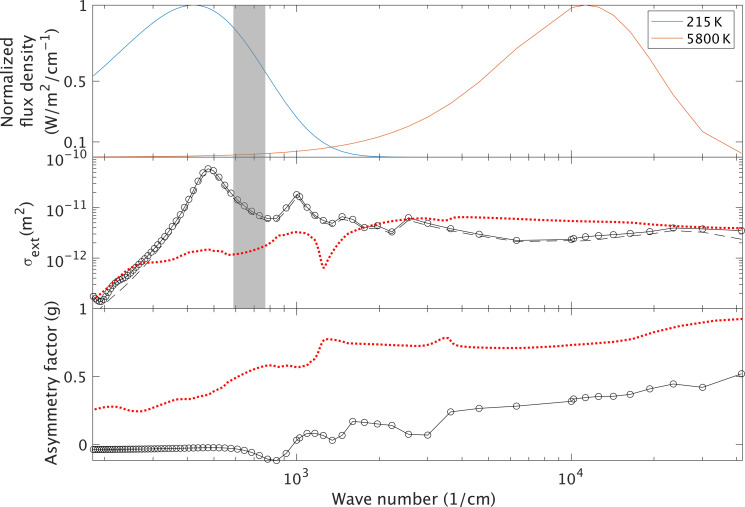
Orientation-averaged optical properties of a 9-μm-long Al nanorod with cross section of 0.16 μm by 0.16 μm, calculated using a 3D finite-difference time-domain approach. **Top**: Planck functions (normalized flux density, W/m^2^/μm) for 215 K (Mars thermal emission now, blue) and 5800 K (insolation, red). For context, the CO_2_ band is overlain (gray shading) at 12 to 16 μm. **Middle**: Black solid line corresponds to total extinction, and black dotted line corresponds to scattering. **Bottom**: Scattering asymmetry. Also shown are wavelength dependence of total extinction and asymmetry factor for natural dust assuming a log-Gaussian particle size distribution centered on 2.5 μm (*48*) (red dotted lines).

Consider a 9-μm-long conductive nanorod (we consider aluminum and iron) with a ~60:1 aspect ratio, not much smaller than commercially available glitter. Finite-difference time domain calculations (Supplementary Text) show that such nanorods, randomly oriented due to Brownian motion (*18*), would strongly scatter and absorb upwelling thermal infrared in the spectral windows and forward-scatter sunlight down to the surface, leading to net warming (Fig. 1 and figs. S1 to S4). Results are robust to changing particle material type, cross-sectional shape, and mesh resolution and change as expected with particle length and aspect ratio (figs. S5 to S8). The calculated thermal infrared scattering is near-isotropic (Fig. 1), which favors surface warming (*19*). Such nanorods would settle >10× more slowly in the Mars atmosphere than natural Mars dust (Supplementary Text), implying that, once the particles are lifted into the air, they would be lofted to high altitude and have a long atmospheric lifetime.

**Fig. 2.**
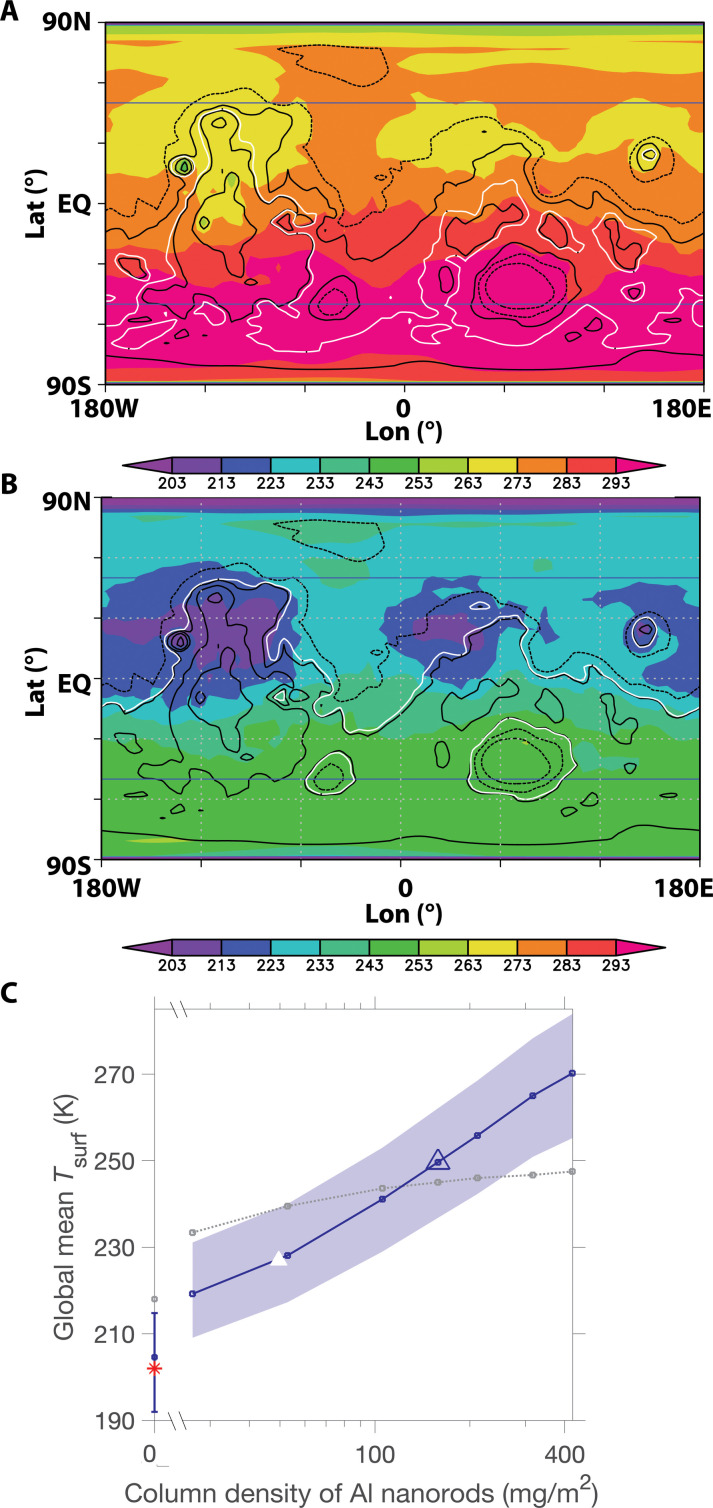
3D model output. Warm-season temperatures (K) (color shading) on (**A**) Mars with addition of ~160 mg/m^2^ of Al nanorods, (**B**) control case. This corresponds to the average surface temperature during the warmest 36° of solar longitude (~70 days) of the year. White contour corresponds to 610 Pa (~6 mbar) mean pressure level. Black contours correspond to topographic elevation in meters (dashed: −5 and −2 km; solid: 0, +2, and +5 km). Blue lines: Approximate latitudinal (equatorward) extent of ice at <1 m depths. Results do not include CO_2_ outgassing from within polar ice, which would cause further warming. (**C**) Dependence of planet-averaged surface warming on Al nanorod column mass. The blue envelope corresponds to the modeled seasonal range in global mean T_surf_. Gray corresponds to 1D results (see the main text for details). Blue line corresponds to 3D results. Blue triangle corresponds to (A), and white triangle marks onset of warm-season temperatures above the freezing point of water at 50°S. Symbols on *y* axis are temperatures for the no-nanorod case, with the red asterisk corresponding to the observed Mars value. Additional details including comparison to Fe nanorods are shown in figs. S10 to S19. EQ, equator.

This motivates calculating surface warming (K) as a function of (artificial) aerosol column density (kilograms per square meter). The Mars Weather Research and Forecasting (MarsWRF) global climate model is suitable for such a calculation (*20*–*22*). Following many previous works (*22*–*26*), we prescribe a layer of aerosol and calculate the resulting steady-state warming (Supplementary Text). Our calculation does not include dynamic transport of aerosol but includes realistic topography, seasonal forcing, and surface thermophysical properties and albedo. The model output (Fig. 2 and figs. S9 to S19) shows that an Al nanorod column density of 160 mg/m^2^ yields surface temperatures and pressures permitting extensive summertime (i.e., the warmest ~70 sols period each year) liquid water in locations with shallow ground ice. This is >5000× more effective, on a warming-per-unit-mass-in-the-atmosphere basis, than the current state of the art (Supplementary Text) (*8*). Temperatures experienced by subsurface ice will be lower due to insulation by soil. Water ice at <1 m depth is almost ubiquitous poleward of ±50° latitude (blue lines in Fig. 2) (*1*). H_2_O ice is present further equatorward (*27*) but is insulated beneath >1-m soil cover and so would not melt unless the annual average surface temperature is raised close to 273 K.

The greenhouse effect depends in part on the temperature difference between the top of the optically thick infrared emitting/absorbing layer and that of the planet surface; higher clouds have a bigger Δ*T* relative to the surface (due to adiabatic cooling) and so give a stronger greenhouse effect. Therefore, the results depend on both artificial-dust-layer-top height and column density (*28*). Table S2 and fig. S19 show the results varying the layer top height between ~35 and ~28 km. The minimum column density for substantial warming (Fig. 2C) can be estimated by setting optical depth in the spectral window (τ_win_) to unity and solving the following expression for column density *M*_c_ (milligrams per square meter) [e.g., (*24*)]

$$\tau_{\text{win}}=3\;Q_{\text{eff}}\;M_{c}/\left(4\;r\;\rho \right)$$ (1)

Here, *Q*_eff_ is the wavenumber–dependent extinction efficiency, ρ is the nanorod particle density (Al: 2.7 g/cc), and *r* is the effective nanorod particle radius (the radius of a sphere of equivalent volume, 0.38 μm). Here, *Q*_eff_ is the ratio of the extinction cross section in the spectral window (about one-half the maximum cross section, i.e., 3 × 10^−11^ m^2^, from Fig. 1) to the geometric cross section of the equivalent sphere and is ≈60. This gives a minimum column density (*M*_c_) of 20 mg/m^2^. At 160 mg/m^2^, the volumetric density of nanorods, 10 cm^−3^, gives a Brownian coagulation timescale (for 0.1- to 10-μm-diameter spheres, for 100% accretion efficiency) ≈ 6 years (*18*). This timescale estimate has substantial uncertainties; for example, actual accretion efficiency may be less, for example because monodisperse particles of uniform composition (e.g., nanorods) may carry similar charges and thus repel each other (*29*). On Mars, particles would be taken up by dry deposition and by transient CO_2_ ice and rereleased to the atmosphere by dust lifting. Initial release (after manufacture) could be from a pipe extending 10 to 100 m above the surface, as Mars turbulent updrafts strengthen with distance from the surface (*30*). For an effective particle lifetime of 10 years, sustaining the warming shown in Fig. 2A requires particle fountaining at an average rate of 30 liters/s (1 liter/s corresponds to the flow of one standard garden sprinkler). Multiyear lifetimes are consistent with one-pass fall-out of ~0.1-μm-diameter particles (*31*), and particle lifetime might be greatly extended if the particles are engineered to self-loft (*32*–*34*), further reducing the sustaining mass flux; however, effective particle lifetime remains a major uncertainty in our model.

As a check on the three-dimensional (3D) results, we ran a 1D model using annual average Mars insolation (Supplementary Text). This predicts 245 K global temperature for ~160 mg/m^2^ Al nanorods (Fig. 2C and figs. S16 and S17). For further increases in nanorod loading, the 1D model predicts lower global temperatures than does the 3D model. This may be due to differences in the vertical temperature structure of the two models (Supplementary Materials). Even in the warmed climate, the south pole is cold enough for seasonal CO_2_ condensation.

Within months of warming Mars, the atmospheric pressure increases by ~20% as CO_2_ ice sublimes, a positive warming feedback. On a warmed Mars, atmospheric pressure will further increase by a factor of 2 to 20 as adsorbed CO_2_ desorbs (*35*), and polar CO_2_ ice (*36*) is volatilized on a timescale that could be as long as centuries. This will further increase the area that is suitable for liquid water (*6*). However, raising Mars’ temperature, by itself, is not sufficient to make the planet’s surface habitable for oxygenic photosynthetic life: Barriers remain (*7*). For example, Mars’ sands have ~300 parts per million by weight (ppmw) nitrates (*37*), and Mars’ air contains very little O_2_, as did Earth’s air before the arrival of cyanobacteria. Remediating perchlorate-rich soil might require bioremediation by perchlorate-reducing bacteria, which yield molecular oxygen as a natural by-product (*38*).

## DISCUSSION

The results from this relatively simple workflow are subject to several uncertainties that motivate more sophisticated modeling. As one of several examples, modeling of coupled dust flow and ice nucleation on Mars is currently at an early stage (*39*). Modeling the effect of nanorods as ice nuclei—which could either be a positive or a negative feedback, depending on the size and altitude of the resulting water ice cloud particles and their precipitation efficiency—is additional motivation to study this coupling. A thin coating on the nanorods could alter their hydrophobicity level, and potentially the ice nucleation, and might also protect against oxidation. The optimal location(s) for particle fountaining require further research. Release into the ascending limb of the Hadley cell should allow dispersal in both hemispheres. The radiative effect of water vapor feedback is unambiguously positive. Tests varying nanorod size, composition, and shape suggest that further improvements to warming effectiveness are possible (figs. S7 and S8). For example, extinction efficiency decreases approximately linearly with rod radius, but mass decreases quadratically with rod radius.

With the caveats above in mind, Fig. 2C allows a first estimate of how much surface material would be needed to supply the fountains. For surface material density of 2 g/cc and Al_2_O_3_ content of ~10 wt % [e.g., (*40*)], raising the surface temperature to that shown in Fig. 2A over 10 years would require processing 2 × 10^7^ m^3^/year of surface material to obtain 7 × 10^5^ m^3^/year of metal, corresponding to a prismatic mine of half-width of 350 m and a side-wall slope of 20°, lengthening by 250 m per year. This is much easier than the current state of the art (*8*), because fluorine is only infrequently detected by rovers (*41*) and is present in most Mars meteorites only at 15- to 90-ppmw concentrations (*42*). However, even this reduced material processing demand still corresponds to 1 × 10^−3^ of Earth’s metal production, and this defines a major manufacturing problem that remains to be solved. Processing of surface material into nanoparticles might use lenses or mirrors to concentrate sunlight for vacuum evaporation, followed by colloidal growth. Synthetic biology (e.g., magnetite nanorods) is a possible alternative (*43*). Metal 3D printing of parts [e.g., Relativity’s Stargate; (*44*)] and/or assembly on Mars might reduce launch costs. Because of their <2-nm width, carbon nanomaterials might warm Mars more effectively than the nanoparticles shown in Figs. 1 and 2. For example, graphene’s density is 0.77 mg/m^2^, and graphene nanodisks that are ~10^2^-nm diameter have strong mid-infrared resonances (*45*).

**Fig. 3.**
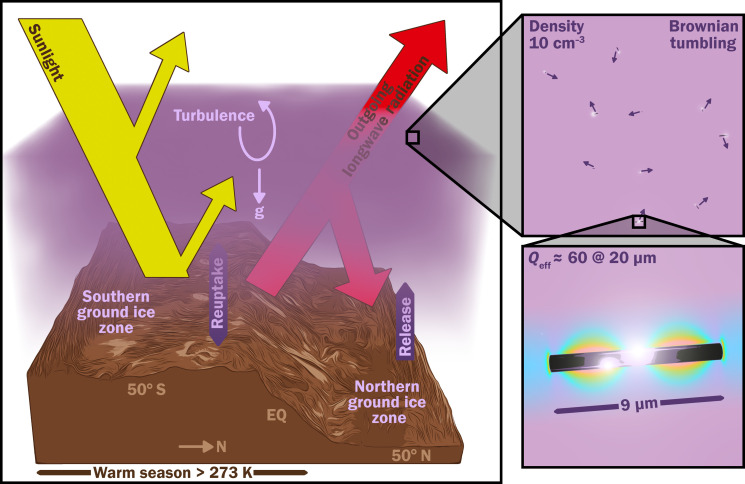
The proposed nanoparticle warming method. Figure credit: Aaron M. Geller, Northwestern, Center for Interdisciplinary Exploration and Research in Astrophysics + IT-RCDS

Although nanoparticles could warm Mars (Figs. 2C and 3), both the benefits and potential costs of this course of action are now uncertain. For example, in the unlikely event that Mars’ soil contains irremediable compounds toxic to all Earth-derived life (this can be tested with Mars Sample Return), then the benefit of warming Mars is nil. On the other hand, if a photosynthetic biosphere can be established on the surface of Mars, perhaps with the aid of synthetic biology, then that might increase the Solar System’s capacity for human flourishing. On the cost side, if Mars has extant life, then study of that life could have great benefits that warrant robust protections for its habitat. More immediately, further research into nanoparticle design and manufacture coupled with modeling of their interaction with the climate could reduce the expense of this method. Examples include Mars pressure wind tunnel experiments for nanorod and dust reuptake and release rate from realistic rough surfaces (including icy surfaces) and mesoscale/large-eddy simulation modeling of nanorod dispersal and lofting. In addition to the nanorod warming option and the no-action option, cost-benefit calculations should also consider local warming methods, such as silica aerogel tiling (*9*).

More work is needed on the very long-term sustainability of a warmed Mars. Atmospheric escape to space would take at least 300 million years to deplete the atmosphere at the present-day rate (*46*). However, if the ground ice observed at meters to tens of meters depth is underlain by empty pore space, then excessive warming over centuries could allow water to drain away, requiring careful management of long-term warming. Subsurface exploration by electromagnetic methods could address this uncertainty regarding how much water remains on Mars deep underground (*47*).

The efficiency of nanoparticle warming suggests that any entity with the goal of strong planet-scale warming would use this approach. This suggests polarization as a technosignature for cold terrestrial worlds with geodynamos.

Nanoparticle warming, by itself, is not sufficient to make the planet’s surface habitable again. Nevertheless, our study suggests that nanoparticle warming may be of interest to the nanophotonics and planetary science communities, among others, and that further investigation might be fruitful.

## MATERIALS AND METHODS

### Calculation of optical properties of nanorods

We carried out finite-difference time-domain simulations (FDTD: 3D Electromagnetic Simulator). Optical properties are obtained at 75 wavelengths, approximately log-uniformly spaced from 0.24 to 55 μm (table S1). Additional details are given in the Supplementary Materials.

### Calculation of the surface warming effect of the nanorods using 1D climate model

Our single-column radiative-convective climate model (RCM) subdivides atmospheres into multiple vertical log layers (we implement 201 layers here) that extend from the ground to the top of the atmosphere (1 × 10^−5^ bar here) [e.g., (*25*)]. The RCM has 55 infrared and 38 solar spectral intervals and applies a standard moist convective adjustment. Additional details are given in the Supplementary Materials.

### Calculation of the surface warming effect of the nanorods using 3D climate model

We use the MarsWRF code (*20*–*22*), with a horizontal resolution of 5.625° × 3.75°, corresponding to a grid of 64 points in longitude × 48 points in latitude. A 40-layer vertical grid is used. A prescribed natural dust aerosol distribution is imposed, giving an average dust optical depth of 0.20 (peaking at 0.43 during southern summer). Additional details are given in the Supplementary Materials.

## Supplementary Materials

### This PDF file includes:

Supplementary Text Figs. S1 to S19 Tables S1 to S3 References
